# DOX-Vit D, a Novel Doxorubicin Delivery Approach, Inhibits Human Osteosarcoma Cell Proliferation by Inducing Apoptosis While Inhibiting Akt and mTOR Signaling Pathways

**DOI:** 10.3390/pharmaceutics10030144

**Published:** 2018-09-04

**Authors:** Zaid H. Maayah, Ti Zhang, Marcus Laird Forrest, Samaa Alrushaid, Michael R. Doschak, Neal M. Davies, Ayman O. S. El-Kadi

**Affiliations:** 1 Faculty of Pharmacy and Pharmaceutical Sciences, University of Alberta, Edmonton, AB T6G 2E1, Canada; almaayah@ualberta.ca (Z.H.M.); mdoschak@ualberta.ca (M.R.D.); ndavies@ualberta.ca (N.M.D.); 2 Cardiovascular Research Centre, Department of Pediatrics and Medicine, Mazankowski Alberta Heart Institute, Faculty of Medicine and Dentistry, University of Alberta, Edmonton, AB T6G 2E1, Canada; 3 Department of Pharmaceutical Chemistry, School of Pharmacy, University of Kansas, Lawrence, KS 66047, USA; tzh217@gmail.com (T.Z.); lforrest@ku.edu (M.L.F.); 4 Department of Pharmaceutical Chemistry, Faculty of Pharmacy, Kuwait University, Safat 13110, Kuwait; samaa.alrushaid@hsc.edu.kw

**Keywords:** doxorubicin, MG63, Vitamin D, DOX-Vit D

## Abstract

Doxorubicin (DOX) is a very potent and effective anticancer agent. However, the effectiveness of DOX in osteosarcoma is usually limited by the acquired drug resistance. Recently, Vitamin D (Vit-D) was shown to suppress the growth of many human cancer cells. Taken together, we synthesized DOX-Vit D by conjugating Vit-D to DOX in order to increase the delivery of DOX into cancer cells and mitigate the chemoresistance associated with DOX. For this purpose, MG63 cells were treated with 10 µM DOX or DOX-Vit D for 24 h. Thereafter, MTT, real-time PCR and western blot analysis were used to determine cell proliferation, genes and proteins expression, respectively. Our results showed that DOX-Vit D, but not DOX, significantly elicited an apoptotic signal in MG63 cells as evidenced by induction of death receptor, Caspase-3 and BCLxs genes. Mechanistically, the DOX-Vit D-induced apoptogens were credited to the activation of p-JNK and p-p38 signaling pathway and the inhibition of proliferative proteins, p-Akt and p-mTOR. Our findings propose that DOX-Vit D suppressed the growth of MG63 cells by inducing apoptosis while inhibiting cell survival and proliferative signaling pathways. DOX-Vit D may serve as a novel drug delivery approach to potentiate the delivery of DOX into cancer cells.

## 1. Introduction

Osteosarcoma (OS) is one of the most widespread and lethal forms of childhood primary bone cancer [1]. In Canada, OS accounts for about 5% of all tumors in pediatric patients with an incidence rate of 8 cases per million each year especially in adolescents [2,3]. Despite the substantial progress in chemotherapies against OS, the mortality rate of OS patients has not been changed significantly due to chemoresistance and other factors [4].

One of those standard therapies for OS is doxorubicin (DOX), an effective anthracycline antibiotic [5]. The combination of DOX with other chemotherapeutic agents such as cisplatin, ifosfamide and methotrexate cured 60–76% of newly diagnosed non-metastatic OS [6]. Although DOX has improved survival rates in cancer patients, the effectiveness of DOX in OS is usually limited by the acquired drug resistance. This resistance is dose-dependent and may develop gradually within a month or years after the treatment initiation. Though the specific mechanism of chemoresistance associated with DOX is still unclear, several reports have demonstrated that drug inactivation, increased DNA damage repair, disturbances in intracellular drug transport and evasion of apoptosis could play a role in the chemoresistance [7].

Numerous epidemiological reports have suggested a strong association between Vitamin D (Vit-D) and cancer risk [8,9]. The deficiency of Vit-D has been reported to contribute to the development of tumors whereas, higher intake of Vit-D was accompanied by a lower incidence of cancer disease [10,11]. Experimental studies using cancer cells or tumors in mice have shown that Vit-D exhibited antitumor activities through the induction apoptosis in addition to the inhibition of cell proliferation and differentiation [12,13].

Vit-D can be classified naturally into animal-based Vit-D3 and plant-based Vitamin D2. Vit-D3, cholecalciferol, is synthesized by the mammalian skin after exposure to sunlight then metabolized into its active form, calcitriol, in the liver and kidneys [14]. Upon binding to its receptor, calcitriol activates several signaling pathways that regulate bone metabolism and calcium homeostasis. Of interest, calcitriol was shown to inhibit the growth, proliferation and differentiation of many cancer cell lines such as breast, prostate and colon cancers [15,16]. However, the anticancer activity of calcitriol was associated with significant hypercalcemia that limits its clinical utility [17]. In contrast to Vit-D3, ergocalciferol, Vit-D2, has been reported to exert a low calcemic effect and potent antitumor activity [14,18]. Ergocalciferol, Vit-D2, occurs naturally in plants and it is synthesized from proVit-D2, ergosterol, upon exposure to sunlight [14]. Of particular interest, it has been reported that Vit-D2 enhanced the cytotoxic effect of DOX on human breast and prostate cancer cell lines [19].

In light of the information described above, we hypothesized that by synthesis of DOX-Vit D, a novel DOX derivative, through conjugating Vit-D2 to DOX, the chemoresistance associated with DOX could be mitigated. For this purpose, the current study was designed to (1) investigate the antiproliferative and apoptogenic effects of DOX-Vit D in the human OS cell line, MG63 cells, and (2) explore the possible mechanism(s) involved. Our study provides substantial evidence that DOX-Vit D suppressed the growth of MG63 cells by inducing apoptosis while inhibiting cell survival and proliferative signaling pathways. Our DOX-Vit D conjugate may be of particular importance in drug delivery and may serve as a novel drug delivery approach to potentiate the delivery of DOX into the bone cancer cells.

## 2. Materials and Methods

### 2.1. Materials

Total Akt (t-Akt) rabbit polyclonal, phosphorylated-Akt (P-Akt) rabbit polyclonal, mammalian target of rapamycin C (t-mTOR) goat polyclonal and p-mTOR rabbit polyclonal were bought from Santa Cruz Biotechnology, Inc. (Santa Cruz, CA, USA). Any other materials used in the current study has been described previously [20].

### 2.2. Chemistry

#### 2.2.1. Calciferol-Succinate

**Figure 1:**
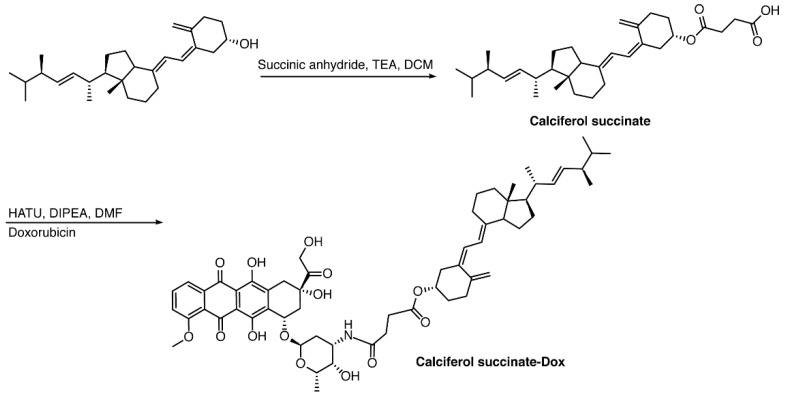
Chemical synthesis of DOX-Vit D.

**Figure 2:**
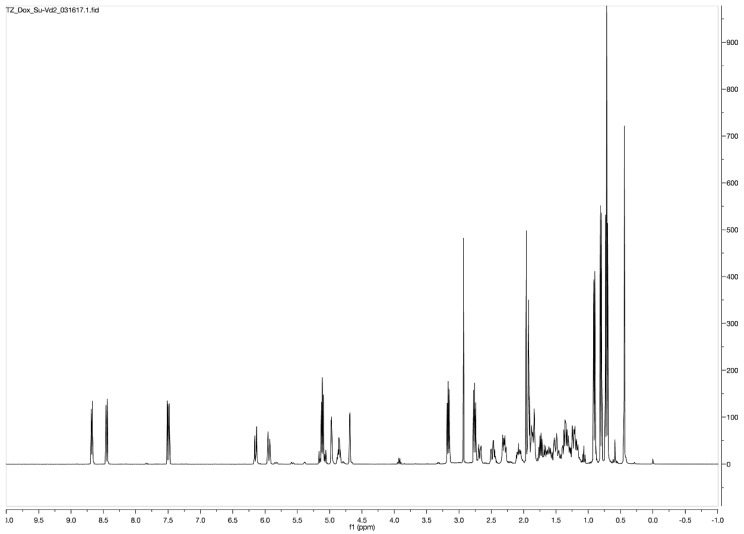
Schematic ^1^HNMR diagram of DOX-Vit D.

In a round bottom flask, 200 mg of calciferol (0.5 mmol) was dissolved in 20 mL of anhydrous dichloromethane (DCM), followed by the addition of 360 mg of succinic anhydride (3.6 mmol, 7.2 eq.) and 500 μL of triethylamine (TEA, 3.6 mmol, 7.2 eq.). The reaction mixture was stirred at ambient temperature under N_2_ for 24 h in the dark. The solution was washed with water three times, and the organic layer was concentrated under reduced pressure. The compound was purified with a Combiflash RF system (hexane/ethyl acetate, 30/70) to obtain a yellowish solid with a yield of 93%. ^1^H NMR (400 MHz, Acetone-d6) *δ* 6.30 (d, *J* = 11.2 Hz, 1H), 6.10 (dt, *J* = 11.3, 1.6 Hz, 1H), 5.35–5.18 (m, 2H), 5.12 (dt, *J* = 2.6, 1.2 Hz, 1H), 4.94 (tt, *J* = 7.8, 3.8 Hz, 1H), 4.84 (d, *J* = 2.6 Hz, 1H), 4.07 (q, *J* = 7.1 Hz, 1H), 2.90 (dd, *J* = 11.9, 4.0 Hz, 1H), 2.69–2.50 (m, 5H), 2.49–2.32 (m, 2H), 2.27–2.16 (m, 1H), 2.16–1.94 (m, 6H), 1.89 (qd, *J* = 6.9, 5.9 Hz, 1H), 1.83–1.65 (m, 4H), 1.66–1.27 (m, 7H), 1.22 (t, *J* = 7.1 Hz, 1H), 1.07 (d, *J* = 6.7 Hz, 3H), 0.96 (d, *J* = 6.9 Hz, 3H), 0.87 (t, *J* = 6.6 Hz, 6H) (Figure 1 and Figure 2).

#### 2.2.2. Calciferol-Succinate-DOX

Calciferol-succinate (230 mg, 0.463 mmol), HATU, 1-[Bis(dimethylamino)methylene]-1H-1,2,3-triazolo[4,5-b]pyridinium 3-oxid hexafluorophosphate (HATU) (211 mg, 1.2 eq.), and Dox-HCl (295 mg. 1.1 eq.) were dissolved in 10 mL of anhydrous *N*,*N*-Dimethylformamide (DMF). To the he mixture was added 400 μL of *N*,*N*-Diisopropylethylamine (DIPEA). The solution was stirred at ambient temperature under N_2_ for 24 h in the dark. The crude mixture was dried under reduced pressure, and purified with a Combiflash RF system (hexane/ethyl acetate, 50/50) to afford product as an orange solid with a yield of 60%. ^1^H NMR (400 MHz, Acetone-d6) *δ* 8.68 (dd, *J* = 4.4, 1.4 Hz, 1H), 8.50–8.37 (m, 1H), 7.56–7.38 (m, 1H), 6.14 (d, *J* = 11.2 Hz, 1H), 5.94 (dd, *J* = 11.2, 1.8 Hz, 1H), 5.23–5.05 (m, 2H), 4.97 (dq, *J* = 3.6, 2.2, 1.8 Hz, 1H), 4.86 (tt, *J* = 7.7, 3.8 Hz, 1H), 4.69 (d, *J* = 2.7 Hz, 1H), 3.22–3.09 (m, 2H), 2.84–2.71 (m, 2H), 2.71–2.63 (m, 1H), 2.54–2.39 (m, 2H), 2.37–2.24 (m, 2H), 2.08 (dddd, *J* = 12.3, 5.9, 4.7, 3.5 Hz, 2H), 1.98–1.79 (m, 8H), 1.78–1.55 (m, 4H), 1.55–1.42 (m, 3H), 1.42–1.10 (m, 9H), 0.95–0.86 (m, 4H), 0.80 (d, *J* = 6.8 Hz, 4H), 0.72 (t, *J* = 6.7 Hz, 7H). ESI (*m*/*z*); calculated for C_59_H_79_N_2_O_14_ [M + NH_4_]^+^: 1039.5531; found: 1039.7386 (Figure 1 and Figure 2).

### 2.3. Cell Culture and Treatments

The human osteosarcoma cancer cell line, MG63 cells, (ATCC, Manassas, VA, USA) was maintained according to the ATCC’s instructions.

### 2.4. Effect of DOX and DOX-Vit D on MG63 Cell Proliferation

The effect of DOX and DOX-Vit D on MG63 cell proliferation was determined by measuring the capacity of reducing enzymes to convert 3-[4,5-dimethylthiazol-2-yl]-2,5-diphenyltetrazoliumbromide (MTT) to colored formazan crystals as described previously [21,22]. The percentage of cell proliferation was calculated relative to control wells designated as 100% viable cells using the following formula:

(1) $$\mathrm{cell}\;\mathrm{proliferation}=\left(A_{\mathrm{treated}}\right)/\left(A_{\mathrm{control}}\right)\times 100\%$$

### 2.5. RNA Extraction and cDNA Synthesis

Total RNA was extracted using TRIzol reagent (Invitrogen^®^, Carlsbad, CA, USA) as described previously [20].

### 2.6. Quantification of mRNA Expression by Quantitative Real-Time Polymerase Chain Reaction (Real Time-PCR)

Quantification of specific gene expression was performed by real time-PCR using ABI Prism 7500 System (Applied Biosystems, Foster City, CA, USA) as previously described [23]. Human primers sequences and probes for Caspase-3, p53, BCLxs, death receptor-4 (DR-4), heme oxygenase-1 (HO-1), NAD(P)H:quinone oxidoreductase-1 (NQO-1)and β-actin are illustrated in Table 1. These primers were purchased from Integrated DNA Technologies (IDT, Coralville, IA, USA).

### 2.7. Determination of Reactive Oxygen Species (ROS) Production

ROS was measured fluorometrically using 2,7-dichlorofluorescein diacetate (DCF-DA) assay as described previously [24]. Briefly, MG63 cells were treated for 24 h with 10 µM DOX-Vit D. Thereafter, cells were washed with PBS before incubated for 30 min in fresh media containing 10 μM DCF-DA. The fluorescence was directly measured using excitation and emission wavelengths of 485 and 535 nm, respectively, the Bio-Tek Synergy H1Hybrid Multi-Mode Microplate Readers (Bio-Tek Instruments, Winooski, VT, USA).

### 2.8. Protein Extraction from MG63 Cells

MG63 cells were treated for 24 h with 10 µM DOX-Vit D or DOX, then the total cellular protein was extracted from the cells as described previously [20].

### 2.9. Immuno Blot Analysis

Cell lysates were analyzed by SDS-PAGE and immunoblotting were performed as described previously [20].

### 2.10. Determination of MAPKs Signaling Pathway

The protein phosphorylation of MAPKs was measured using the PhosphoTracer MAPK ELISA Kit (Abcam, Cambridge, UK) according to manufacturer’s instructions and as described previously [20].

### 2.11. Extration of Nuclear Protein

MG63 cells were treated for 2 h with 10 µM DOX-Vit D or DOX, then the nuclear protein was extracted from the cells as described previously [20,25].

### 2.12. Determination of NF-κB Binding Activity

The NF-κB binding activity was determined using NF-κB Assay Chemiluminescent Kit (Millipore, Schwalbach/Ts., Germany, #70-660) according to the manufacturer’s protocol as described previously [26].

### 2.13. Statistical Analysis

Results are shown as mean ± SEM. Statistical analysis was carried out using SigmaPlot^®^ for Windows (Systat Software, Inc., San Jose, CA, USA). One-way analysis of variance (ANOVA) followed by Tukey-Kramer multiple comparison tests or unpaired two-sided student *t*-test was carried out. A probability value obtained less than 0.05 is considered significant.

## 3. Results

### 3.1. Physiochemical Properities of DOX-Vit D in Comaprison to DOX

Given that the main purpose of the current study is to improve the lipopilicity of DOX, we investigated the physiochemical properties of DOX-Vit D in comparison to DOX using ACD iLab and VCCLAB software (https://www.acdlabs.com/resources/ilab/) as described previously [27]. Perhaps the better predictor of lipophilicity is the distribution coefficient at pH 7.4 (LogD_7.4_) since it considers the ionizable group at certain pH in addition to the estimated partition coefficient (Log*P*). Of interest, Table 2 shows that DOX-Vit D has clear higher predicted values of Log*P* and LogD_7.4_ in comparison to DOX. This was consistent with a low predicted water solubility of DOX-Vit D (0.0029 μg/mL) in comparison to DOX (0.49 mg/mL) and a higher Log*S* value for DOX-Vit D. Together, it is reasonable to assume that our novel DOX derivative, DOX-Vit D, is more lipophilic than DOX.

### 3.2. Effect of DOX and DOX-Vit D on MG63 Cells Proliferation

**Figure 3:**
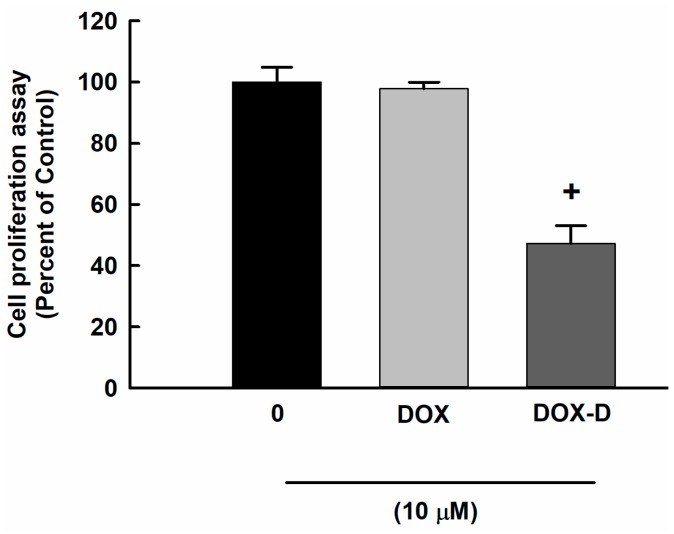
Effect of DOX and DOX-Vit D on MG63 cells proliferation. MG63 cells were exposed to 10 μM DOX and DOX-Vit D for 24 h. Thereafter, the cell proliferation was determined using MTT assay. The results are presented as the mean ± SEM (*n* = 6). ^+^ *p* < 0.05 compared to control.

To determine the cytotoxic effect of DOX and DOX-Vit D on OS, MG63 cells were exposed to 10 μM DOX and DOX-Vit D for 24 h. Thereafter, the cell proliferation was determined using MTT assay. Our results showed that a 10 μM DOX did not significantly affect cell proliferation at 24 h (Figure 3). However, 10 μM DOX-Vit D significantly decreased the cell proliferation by approximately 50% in comparison to control (Figure 3).

### 3.3. Effect of DOX and DOX-Vit D on Proapoptotic Genes

**Figure 4:**
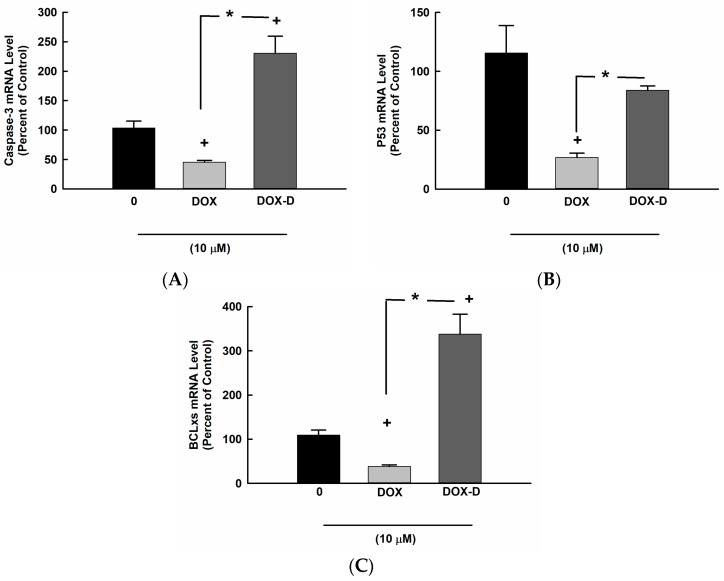
Effect of DOX and DOX-Vit D on proapoptotic genes. MG63 cells were treated for 24 h with 10 µM DOX and DOX-Vit D. Thereafter, total RNA was isolated using TRIzol reagent, and the mRNA levels of (**A**) Caspase-3, (**B**) p53 and (**C**) BCLxs were quantified using real time-PCR and normalized to a β-actin housekeeping gene. The results are presented as the mean ± SEM (*n* = 6). *^+^ p* < 0.05 compared to control. * *p* < 0.05 compared to DOX.

To investigate whether the inhibitory effect of DOX-Vit D on MG63 cell proliferation and growth is an apoptosis-dependent mechanism, MG63 cells were treated for 24 h with 10 µM DOX and DOX-Vit D. Thereafter, the mRNA levels of proapoptotic genes, Caspase-3, p53 and BCLxs, were determined by real time-PCR. Figure 4 shows that DOX-Vit D caused a significant induction of Caspase-3 and BCLxs genes expression by approximately 250% and 400%, respectively, in comparison to control. On the other hand, DOX significantly decreased the expression of Caspase-3, BCLxs and P53 by about 50%, 20% and 30%, respectively, in comparison to control.

In light of our findings, DOX-Vit D seems to inhibit the growth of MG63 cells through an apoptosis-dependent mechanism. Next, we questioned whether the DOX-Vit D elicited an apoptotic signal in MG63 cells is mediated extrinsically through the activation of death receptor and/or intrinsically by the induction of oxidative stress. Therefore, a series of independent experiments were conducted as follows.

### 3.4. Effect of DOX and DOX-Vit D on the Expression of DR-4

**Figure 5:**
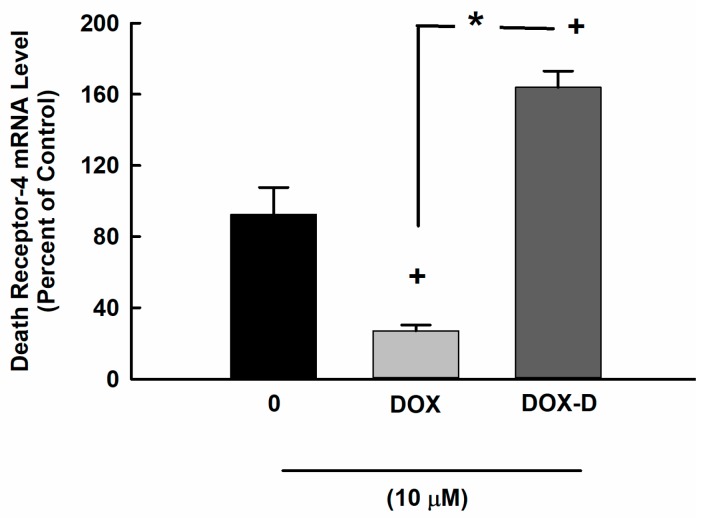
Effect of DOX and DOX-Vit D on the expression of DR-4. MG63 cells were treated for 24 h with 10 µM DOX and DOX-Vit D. Thereafter, total RNA was isolated using TRIzol reagent, and the mRNA level of DR-4 was quantified using real time-PCR and normalized to a β-actin housekeeping gene. The results are presented as the mean ± SEM (*n* = 6). ^+^ *p* < 0.05 compared to control. ^*^ *p* < 0.05 compared to DOX.

In order to determine the capacity of DOX and DOX-Vit D to modulate the expression of DR-4 mRNA, MG63 cells were treated for 24 h with 10 µM DOX and DOX-Vit D. Thereafter, the mRNA levels of DR-4 was determined by real time-PCR. Figure 5 shows that treatment of MG63 cell with DOX-Vit D caused a significant induction of DR-4 by about 170% in comparison to control. On the other hand, DOX significantly inhibit the expression of DR-4 by approximately 60% in comparison to control.

### 3.5. Effect of DOX and DOX-Vit D on the Oxidative Stress

**Figure 6:**
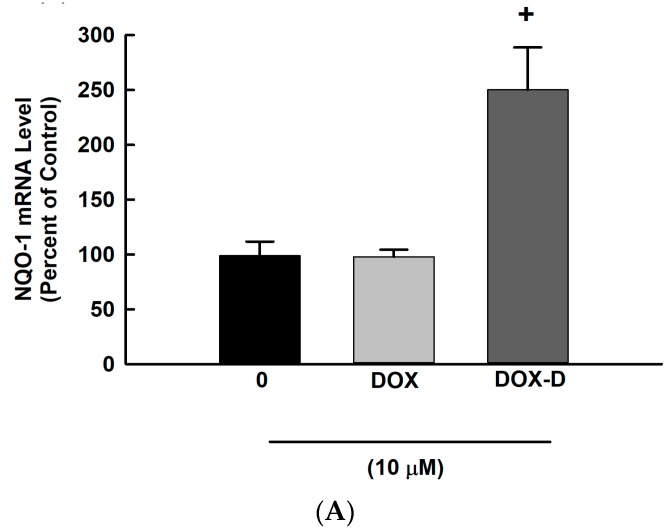

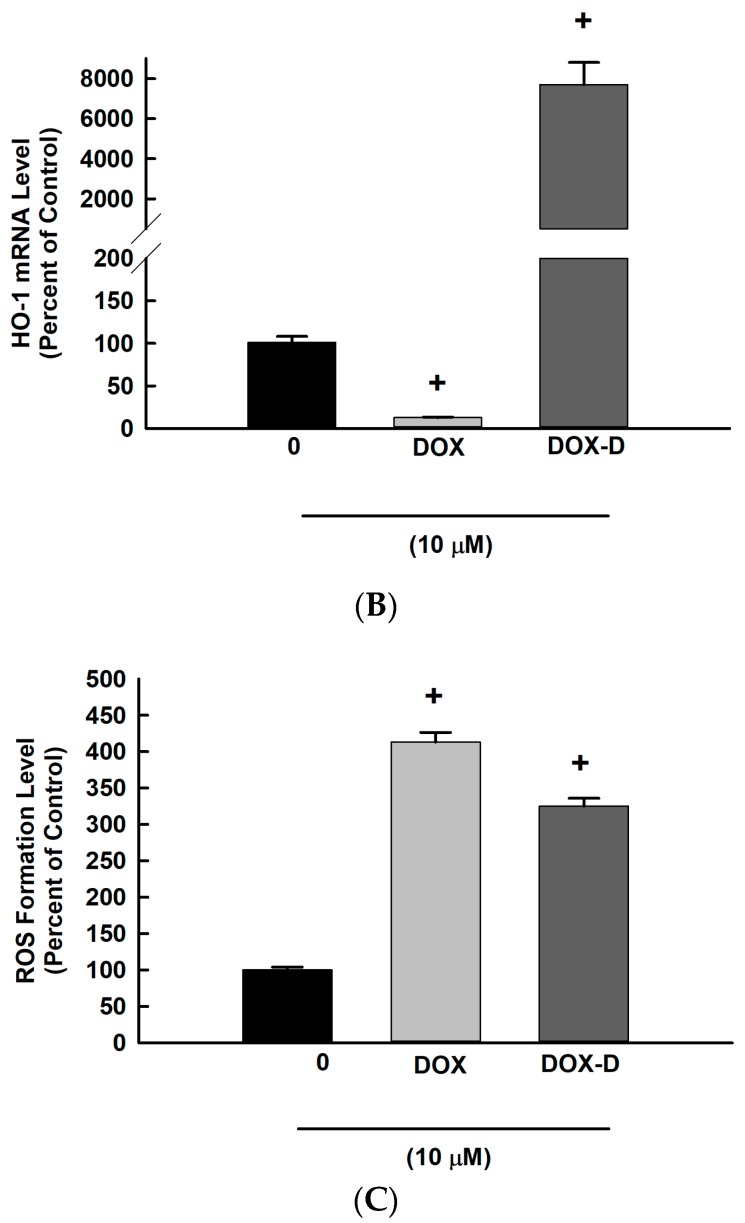
Effect of DOX and DOX-Vit D on the oxidative stress. MG63 cells were treated for 24 h with 10 µM DOX and DOX-Vit D. Thereafter, total RNA was isolated using TRIzol reagent, and the mRNA levels of (**A**) NQO-1 and (**B**) HO-1 were quantified using real time-PCR and normalized to a β-actin housekeeping gene. (**C**) MG63 cells were treated for 24 h with 10 µM DOX and DOX-Vit D then, cells were incubated with DCF-DA (10 μM) for 1 h. DCF formation was measured fluorometrically using excitation/emission wavelengths of 484/535  nm. The results are presented as the mean ± SEM (*n* = 6). ^+^ *p* < 0.05 compared to control.

The involvement of intrinsic apoptotic pathway was addressed by two approaches. Firstly, we determined the effect of DOX-Vit D and DOX on the mRNA expression of oxidative stress markers. Figure 6 shows that treatment of cells with 10 µM DOX-Vit D caused a significant induction of NQO-1 and HO-1 by approximately 250% and 6000%, respectively, in comparison to control. In contrast, DOX significantly inhibited the expression of HO-1 by about 70%, whereas no significant changes were observed with NQO-1 (Figure 6A,B).

The second approach was to investigate the effect of DOX and DOX-Vit D on the generation of ROS using DCF assay. The incubation of MG63 cells with DOX and DOX-Vit D for 24 h caused a significant increase in the formation of ROS by about 400% and 350%, respectively, in comparison to control (Figure 6C).

Our findings suggest an involvement of both extrinsic and intrinsic pathways in the induction of proapoptotic genes by DOX-Vit D. The induction of the aforementioned pathways are known to trigger apoptosis through the activation of MAPK signaling pathway. Thus, we have investigated whether or not DOX-Vit D induces proapoptotic genes through MAPK signaling pathway.

### 3.6. Effect of DOX and DOX-Vit D on MAPK Signaling Pathway

**Figure 7:**
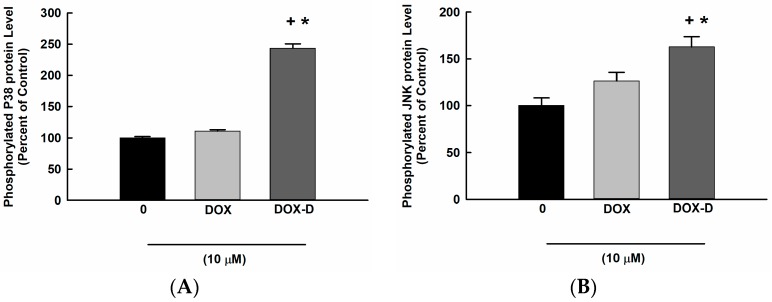

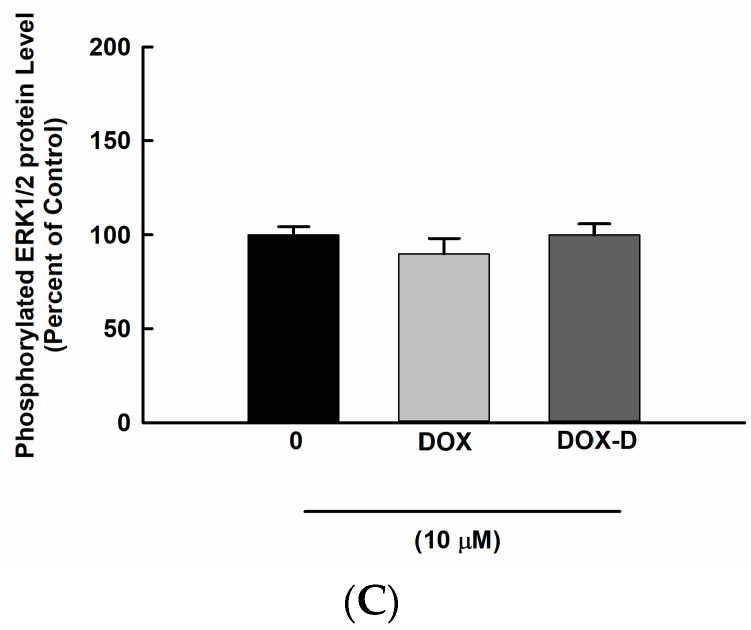
Effect of DOX and DOX-Vit D on MAPK signaling pathway. MG63 cells were treated for 24 h with 10 µM DOX and DOX-Vit D. Thereafter, MAPKs protein phosphorylation was determined in cytoplasmic protein extracts using the PhosphoTracer (**A**) p38 MAPK (pT180/Y182) (**B**) JNK1/2/3 (pT183/Y185) (**C**) ERK1/2 (pT202/Y204) Elisa Kit (Abcam, Cambridge, UK). The results are presented as the mean ± SEM (*n* = 6). ^+^ *p* < 0.05 compared to control. ^*^ *p* < 0.05 compared to DOX.

To assess the role of MAPK signaling pathway on the DOX-Vit D mediated induction of proapoptotic genes, MG63 cells were treated with 10 µM DOX-Vit D and DOX. Thereafter, phosphorylated MAPK levels were determined using a commercially available kit. Figure 7 shows that incubation of the cells with 10 µM of DOX-Vit D but not DOX significantly induced phosphorylation of p38 and JNK by approximately 250% and 160%, respectively, in comparison to control.

**Figure 8:**
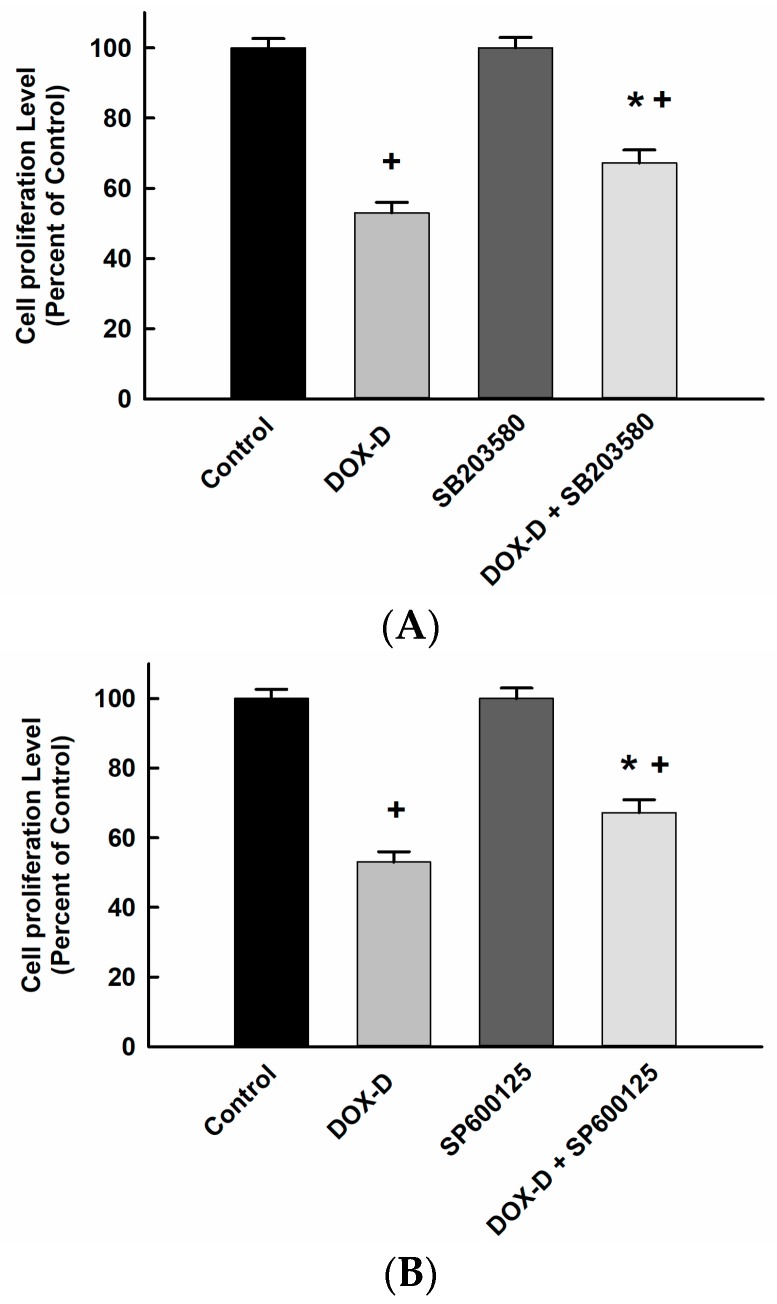
Effect of MAPK inhibitors on DOX-Vit D-induced cytotoxicity. MG63 cells were treated with p38 inhibitor, SB203580, and JNK inhibitor, SP600125, in the presence and absence of 10 µM DOX-Vit D. Thereafter, the cell proliferation was determined using MTT assay. The results are presented as the mean ± SEM (*n* = 6). ^+^ *p* < 0.05 compared to control. ^*^ *p* < 0.05 compared to DOX-Vit D.

To further confirm whether activation of the MAPK pathways is required for the apoptotic cell death mediated by DOX-Vit D, MG63 cells were treated with 10 µM p38 inhibitor, SB203580, and JNK inhibitor, SP600125, in the presence and absence of DOX-Vit D. Thereafter, the cells proliferation were measured using MTT assay. Figure 8 shows that DOX-Vit D alone caused a significant inhibition of MG63 cell proliferation by about 50% in comparison to control. Importantly, treatment of cells with SB203580 and SP600125 partially but significantly protects the cells against the cytotoxic effect of DOX-Vit D. Our findings suggest that the activation of MAPK is essential for the cytotoxic effect of DOX-Vit D.

In order to examine whether the inhibitory effect of DOX-Vit D on MG63 cell proliferation and growth is also attributed to the suppression of cell survival and proliferation pathways, we have determined the effect of DOX-Vit D on NF-κB, Akt and mTOR signaling pathways.

### 3.7. Effect of DOX and DOX-Vit D on NF-κB Signaling Pathway

**Figure 9:**
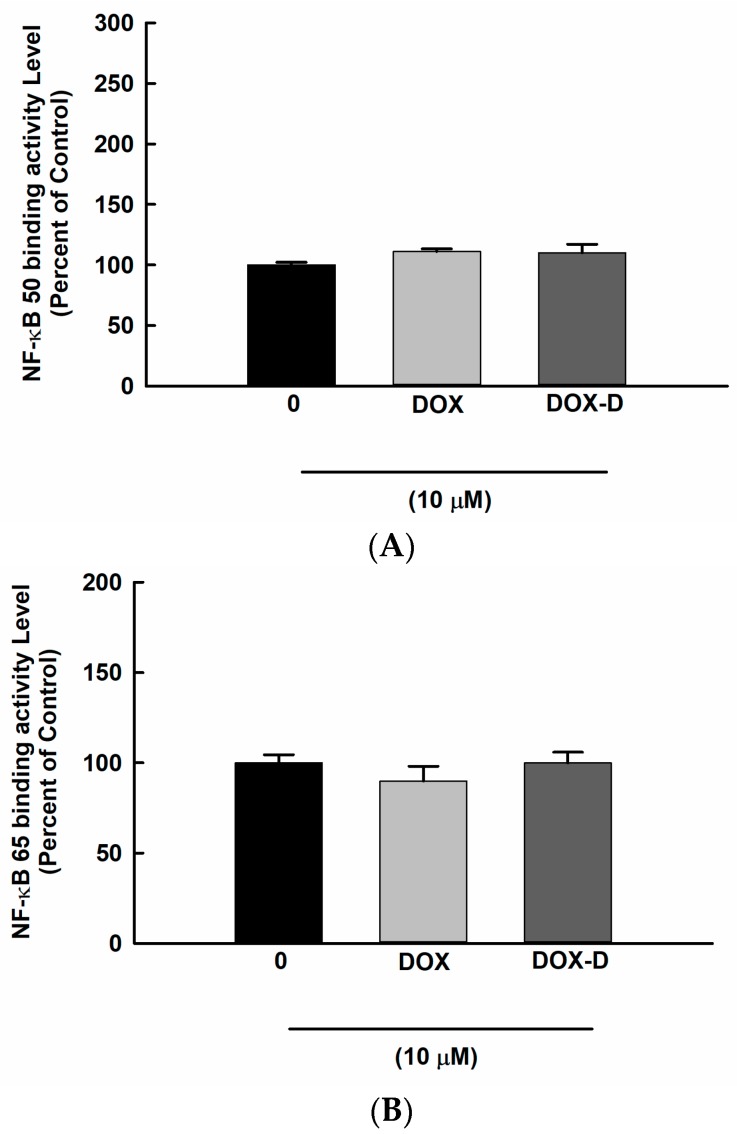
Effect of Effect of DOX and DOX-Vit D on NF-κB signaling pathway. MG63 cells were treated for 24 h with 10 µM DOX and DOX-Vit D. Thereafter, NF-κB binding activity was determined in nuclear extracts using a commercially available kit. The results are presented as the mean ± SEM (*n* = 6).

The basal activity of the NF-κB transcription factor in OS seems to be crucial for their growth or resistance to chemotherapy. Therefore, we have investigated whether DOX-Vit D suppresses MG63 cell growth through the inhibition of NF-κB. For this purpose, MG63 cells were treated with 10 µM DOX and DOX-Vit D. Thereafter, NF-κB binding activity was determined using a commercially available kit. Figure 9 shows that neither DOX nor DOX-Vit D significantly affects the binding activity of NF-κB suggesting an NF-κB-independent mechanism.

### 3.8. Effect of DOX and DOX-Vit D on Akt and mTOR Signaling Pathway

**Figure 10:**
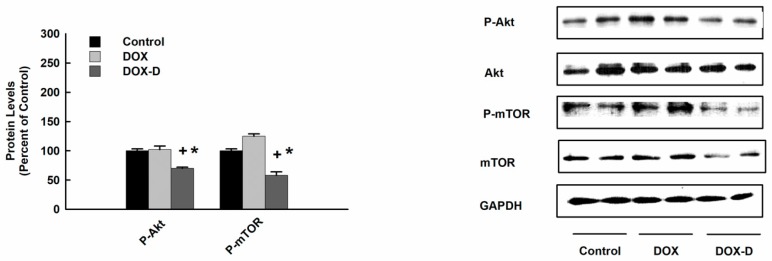
Effect of DOX and DOX-Vit D on Akt and mTOR signaling pathway. MG63 cells were treated for 24 h with 10 µM DOX and DOX-Vit D. Thereafter, total and phosphorylated Akt and mTOR protein expression levels were determined by Western blot analysis and detected using the enhanced chemiluminescence method. The intensity of protein bands was normalized to the signals obtained for GAPDH protein and quantified using ImageJ^®^. The results are presented as the mean ± SEM (*n* = 6). *^+^ p* < 0.05 compared to control. * p < 0.05 compared to DOX.

Since Akt and mTOR pathway promotes cell growth, proliferation and survival, we examined the effect of DOX-Vit D on Akt and mTOR signaling pathway. For this purpose, MG63 cells were treated with 10 µM DOX and DOX-Vit D. Thereafter, Akt and mTOR protein expression levels were determined using Western blot analysis. Figure 10 shows that DOX-Vit D caused a significant inhibition of p-Akt and p-mTOR protein expression by approximately 40% and 50%, respectively, in comparison to control suggesting an Akt/mTOR-dependent inhibition of cell growth by DOX-Vit D. In contrast, DOX did not significantly alter the expression of p-Akt and p-mTOR protein expression.

## 4. Discussion

These investigations provide strong evidence that DOX-Vit D suppresses the growth of human OS, MG63 cell line, through the induction of apoptosis and the inhibition of cell survival and proliferative signaling pathways.

One of the strategies for treating OS and minimizing the development of chemoresistance associated with chemotherapeutic agents includes the induction of apoptosis and/or the attenuation of cell survival and proliferative signaling pathways. Studies using transgenic mice provide direct evidence that overexpression of cell survival pathways and/or disruption of apoptosis promote tumorigenesis, metastasis and contribute to chemoresistance [28,29]. Therefore, the development of a new chemotherapeutic agent that is able to attenuate the proliferation of OS while inducing apoptosis is an urgently needed to overcome chemoresistance.

DOX, a broad-spectrum anthracycline antibiotic, is one of those standard therapies for the treatment of OS [5]. Unfortunately, the effectiveness of DOX in OS is usually limited by the acquired drug resistance that leads to poor prognosis and suboptimal outcomes [30]. Recently, Vit-D has been shown to suppress the growth of many human cancer cells and reverse chemotherapy drug-resistant [30,31]. Taken together, we synthesized DOX-Vit D by conjugating Vit-D to DOX in order to mitigate the chemoresistance associated with DOX. The current study was conducted to investigate the antiproliferative and apoptogenic effects of 10 μM DOX-Vit D in comparison to 10 μM DOX and the possible mechanism(s) involved using the human OS cell line, MG63 cells. The concentration of DOX used in the current study was maintained within the therapeutic range of plasma concentration reported in human. For example, human subjects given a dose of 60–75 mg/m^2^ DOX for the treatment of metastatic cancer had mean plasma concentrations range from 5 and 15 μM and an average half-life of ∼25 h [32,33]. In addition, several in vitro studies on human cancer cells to explore the cytotoxicity of DOX used concentrations range from 1 to 10 μM [32,33].

Initially, we have demonstrated that DOX-Vit D, but not DOX, was able to significantly suppress the MG63 cell proliferation and growth. Notably, the anticancer effect of DOX-Vit D is attributed to the induction of proapoptotic genes Caspase-3 and BCLxs. Activation of the proapoptotic genes plays a crucial role in the initiation of apoptosis through the cleavage of the key cellular proteins resulting in the irreversible commitment to cell death [29,34]. Similar to our observation, it has been reported that calciferol and its chemical derivative, MT19c, induce apoptosis and inhibit the growth and proliferation of many cancer cell lines through the activation of the Caspase-3 enzyme [35,36]. The inhibitory effect of DOX on proapoptotic genes might be attributed to the fact that our MG-63 cells are resistant to DOX. In agreement with our results, it has been shown that 10 μM DOX was neither elicit Caspase-3 activation or apoptosis in DOX resistant MG63 cells [37]. Importantly, promoting apoptosis has been shown to overcome the chemoresistance associated with DOX during the treatment of OS [38].

Apoptosis is known to be elicited by the activation of extrinsic and/or intrinsic signals. These signals are instructing the cells to undergo programmed cell death through the activation of proapoptotic genes [29,34]. Accordingly, we investigated whether the DOX-Vit D-induced apoptosis in MG63 cells is mediated through the extrinsic and/or intrinsic apoptotic pathway. The extrinsic signals induce apoptosis through binding of cell surface death receptors such as TNF/Fas-receptor with its ligand and subsequently activates proapoptotic enzymes [39]. Hence, we have tested whether DOX-Vit D triggers extrinsic apoptotic pathway by measuring the expression of DR-4. In this current study, we found that the induction of DR-4 mRNA in response to DOX-Vit D significantly contributes to the activation of proapoptotic genes. These findings are in agreement with the observation that calciferol increases the activity of proapoptotic genes through the induction of DR [36]. On the other hand, DOX seems to decrease the expression of proapoptotic genes through the downregulation of DR-4.

The intrinsic signal is another pivotal pathway that could initiate apoptosis through the oxidative stress and ROS-dependent mechanism [40]. Oxidative stress and ROS have been considered as a potent inducer of apoptosis [41]. In this context, the involvement of the intrinsic apoptotic pathway in the cytotoxic effect of DOX-Vit D was evidenced by the induction of the oxidative stress markers, NQO-1 and HO-1, in addition to the generation of ROS. In a manner similar to our observations, it has been reported that calciferol treatment results in the accumulation of ROS and subsequently coordinates proapoptotic genes activation [36].

Our findings suggest that DOX-Vit D upregulated the expression of proapoptotic genes through the activation of both extrinsic and intrinsic apoptotic pathways. The induction of the aforementioned pathways are known to trigger apoptosis through the activation of the MAPK signaling pathway. A wealth of information suggests the involvement of MAPK cascades in cell death and survival signaling [42,43]. In particular, it has demonstrated that persistent activation of p38 and JNK promotes apoptosis and cell death [42,43]. Taken together, the possibility that DOX-Vit D would induce apoptosis through the activation of MAPKs could not be ruled out. Thus, the third objective of the current study was to explore the role of DOX-Vit D on the MAPKs signaling pathway. Our results demonstrate that DOX-Vit D, but not DOX, significantly increased the protein expression level of p-p38 and p-JNK whereas no significant changes were observed on p-ERK1/2. The direct evidence for the involvement of p-p38 and p-JNK in the DOX-Vit D-induced cytotoxicity was supported by the observation that p-p38 inhibitor, SB203580, and JNK inhibitor, SP600125, significantly protect against DOX-Vit D-induced cell death suggesting a MAPK-dependent mechanism. The premise of this observation emerges from the finding that calciferol chemical derivative, MT19c, induces apoptosis through a p38 and JNK-dependent mechanism [35].

Apoptosis-mediated cell death might induce the turn off of survival pathways, such as NF-κB and Akt/mTOR pathways, that could otherwise interfere with the apoptotic response. The aberrant activation of those proliferating proteins in OS seems to be crucial for their growth or resistance to chemotherapy [44,45]. Thus, it is imperative to investigate the effect of DOX-Vit D on the aforementioned cell survival and proliferating pathways. Although DOX-Vit D did not significantly affect the binding activity level of NF-κB in MG63 cells, DOX-Vit D significantly downregulated the protein expression level of p-Akt and p-mTOR. In addition, DOX did not significantly alter the p-Akt/p-mTOR proteins expression. These results are in agreement with previous reports on OS showing that high Akt/mTOR activity was associated with poor clinical outcome and chemoresistence associated with DOX [46,47]. On the other hand, everolimus, an mTOR inhibitor, has been shown to decrease drug-induced resistance in OS [48,49]. Our findings not only suggest an Akt/mTOR-dependent inhibition of MG63 cell growth by DOX-Vit D but also support DOX-Vit D as a promising developmental strategy for the treatment of OS resistant to chemotherapy [50].

To reiterate, our results clearly demonstrated that DOX-Vit D, a novel DOX derivative, suppresses MG63 cell growth through the induction of apoptosis and the inhibition of Akt/mTOR signaling pathways. Such observation will raise the potential of developing DOX-Vit D analogues for the treatment of OS resistant to chemotherapy. Given that DOX-VitD has a higher lipophilicity compared to DOX, it is reasonable to assume that DOX-VitD may serve as a novel drug delivery approach to minimize the first-pass effect while increasing lymphatic exposure and ultimately improving overall systemic drug exposure. Additional studies are going to test the toxicity and the kinetic of DOX-VitD in rats. Our preliminary data have shown that DOX-VitD is tolerable upon oral and intravenous administration in rats. Given that our new derivative has a stoichiometry of 1 to 1 ratio (Dox-VitD), it might not necessarily be the most active one. Therefore, we will compare our new derivative with a combination of the free drugs at different ratios in order to confirm the mechanism(s) of action and discriminate additive effects vs synergistic effects.

## Figures and Tables

**Table 1:** Primers sequences used for RT-PCR reactions.

Gene	Forward Primer	Reverse Primer
Caspase-3	GAGTGCTCGCAGCTCATACCT	CCTCACGGCCTGGGATTT
P53	GCCCCCAGGGAGCACTA	GGGAGAGGAGCTGGTGTTG
DR4	AGTACATCTAGGTGCGTTCCTG	GTGCTGTCCCATGGAGGTA
BCLxs	CCCAGAAAGGATACAGCTGG	GCGAT-CCGACTCACCAATAC
HO-1	ATGGCCTCCCTGTACCACATC	TGTTGCGCTCAATCTCCTCCT
NQO-1	CGCAGACCTTGTGATATTCCAG	CGTTTCTTCCATCCTTCCAGG
β-actin	CCAGATCATGTTTGAGACCTTCAA	GTGGTACGACCAGAGGCATACA

**Table 2:** Physicochemical properties of DoxVD vs. Dox and Vitamin D2.

Compound	Doxorubicin (Free Base)	Vitamin D2	DoxVD
Structure	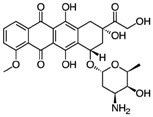	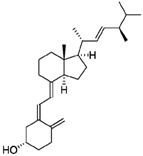	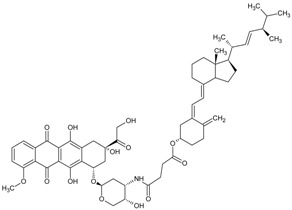
Chemical Formula	C_27_H_29_NO_11_	C_28_H_44_O	C_59_H_75_NO_14_
Molecular Weight (g/mol)	543.53	396.65	1022.22
Log*P* (ACD Chemsketch)	2.82 ± 1.30	9.56 ± 0.27	12.83 ± 1.32
Log*P* (VCCLAB)	1.41	7.59	5.95
Log*P* (experimental, Pubchem)	1.27	7.3	NA
Log D_7.4_ (ACD iLab)	−0.29	7.5	8.68
Solubility H_2_O (ACD iLab)	0.49 mg/mL	0.0018 mg/mL	0.0029 μg/mL
Log*S* (VCCLAB)	−2.67	−5.96	−5.81
Solubility (experimental, drug bank)	2%	0.05 mg/mL	NA
